# The influence of hemodialysis on intracranial pressure waveform in patients with chronic kidney disease: an observational study

**DOI:** 10.1590/1516-3180.2023.0068.R1.07072023

**Published:** 2023-11-27

**Authors:** Mariana Schechtel Koch, Bianca Drewnowski, Cristiane Rickli, Fábio André dos Santos, Gilberto Baroni, José Carlos Rebuglio Vellosa

**Affiliations:** I PhD Candidate, Biologist. Universidade Estadual de Ponta Grossa (UEPG), Ponta Grossa (PR), Brazil.; II PhD Candidate. Pharmacist. Universidade Estadual de Ponta Grossa (UEPG), Ponta Grossa (PR), Brazil.; III PhD. Biomedic, Professor, Centro Universitário Integrado, Campo Mourão (PR), Brazil.; IV PhD. Dentist, Associate Professor, Biological and Health Sciences Division, Universidade Estadual de Ponta Grossa (UEPG), Ponta Grossa (PR), Brazil.; V PhD. Physician, Adjunct Professor, Biological and Health Sciences Division, Universidade Estadual de Ponta Grossa (UEPG), Ponta Grossa (PR), Brazil.; VI PhD. Pharmacist, Associate Professor, Biological and Health Sciences Division, Universidade Estadual de Ponta Grossa (UEPG), Ponta Grossa (PR), Brazil.

**Keywords:** Intracranial pressure, Kidney diseases, Renal dialysis, Glomerular filtration rate, Intracranial hypertension, Nervous system diseases, Neurological disorders, Intracranial compliance, Intracranial pressure waveform

## Abstract

**BACKGROUND:**

Among the complications related to chronic kidney disease (CKD), those of a neurological nature stand out, and for a better quality of life for patients, the diagnosis and treatment of these complications is fundamental.

**OBJECTIVES:**

This study aimed to assess the effect of hemodialysis on intracranial pressure waveform (ICPw) in patients with chronic kidney disease undergoing hemodialysis and those who are not yet undergoing substitutive therapy.

**DESIGN AND SETTING:**

An observational study was conducted in two stages at a kidney replacement therapy center in Brazil. The first was a longitudinal study and the second was a cross-sectional study.

**METHODS:**

Forty-two patients on hemodialysis were included in the first stage of the study. In the second stage, 226 participants were included. Of these, 186 were individuals with chronic kidney disease (who were not undergoing substitutive therapy), and 40 did not have the disease (control group). The participants’ intracranial compliance was assessed using the non-invasive Brain4care method, and the results were compared between the groups.

**RESULTS:**

There was a significant difference between the hemodialysis and non-hemodialysis groups, with the former having better ICPw conditions.

**CONCLUSIONS:**

Hemodialysis influenced the improvement in ICPw, probably due to the decrease in the patients’ extra-and intracellular volumes. Furthermore, ICPw monitoring can be a new parameter to consider when defining the moment to start substitutive therapy.

## INTRODUCTION

Chronic kidney disease (CKD) has become one of the main causes of death and suffering in the 21^st^ century, affecting approximately 10% of the worldwide population, accounting for approximately 843.6 million ill individuals, with a higher prevalence among older adults, women, racial minorities, and those with diabetes mellitus and hypertension.^1^

**Figure 1:**
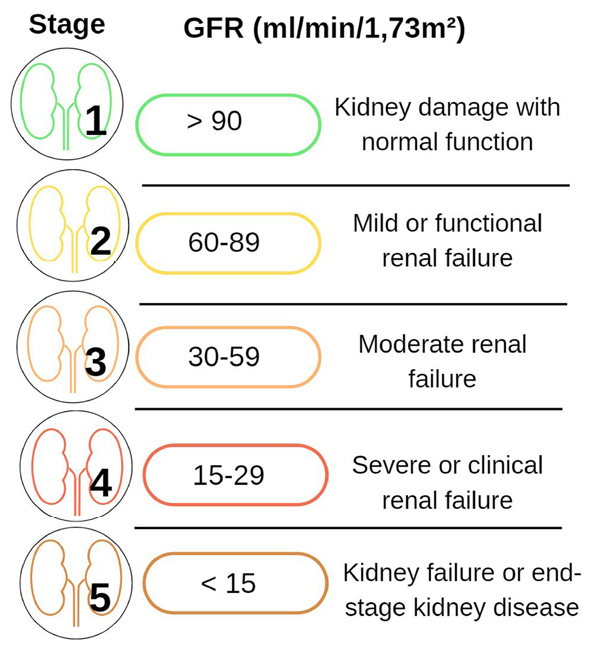
Stages of chronic kidney disease based on the glomerular filtration rate.

Kidney disease has become a public health concern due to its increasing prevalence and high treatment costs for the public. In clinical terms, kidney disease is characterized the loss of kidney function occurs over time. Diagnosis is based on the mean glomerular filtration rate (GFR) (GFR < 60 mL/min/1.73 m^2^ for ≥ 3 months), and the presence of kidney damage is determined through biopsy or other markers of kidney damage.^2^ The classification of the progression of this disease is based mainly on the GFR, and five stages have been established, as shown in Figure 1. The prevalence of the initial stages of chronic kidney disease is significantly higher than that of end-stage kidney disease (ESKD).^3^ The consequences of the illness, besides the loss of kidney function, include cardiovascular disease and premature death,^3^ in which the risk of death due to a cardiovascular event is higher than that of requiring hemodialysis or a transplant. Approximately 4 million people worldwide depend on kidney replacement therapy, of which 89% undergo hemodialysis.^2^ The complications related to hemodialysis are common. Some are even expected due to the hemodialysis process itself, in which the patient bears hours of extracorporeal blood flow, forced ultrafiltration, and exposure to large quantities of dialysate. Unexpected complications include infectious diseases, mineral metabolism disorders, and neurological complications, such as hemodialysis imbalance syndrome.^4^

Neurological complications may be responsible for the incapacity and mortality of patients with CKD and may affect both dialytic and pre-dialysis patients. Additionally, they may affect both the central and peripheral nervous systems and are frequently neglected and rarely acknowledged. Laboratory tests, imaging studies, and neurophysiological tests are some of the tools available for diagnosis. Treatment involves a multifactorial approach, and prognosis depends on the availability of treatment and its precocious start.^5^

**Figure 2:**
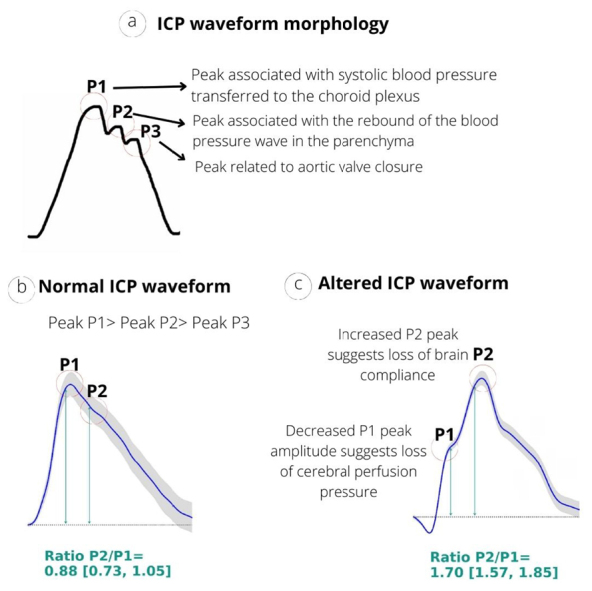
(a) Morphology of an intracranial pressure wave composed of three peaks; (b) Morphology of a normal intracranial pressure wave; (c) Morphology of an intracranial pressure wave with alteration.

A non-invasive intracranial pressure waveform (ICPw) monitoring tool was developed by the Brazilian company Brain4care Inc. (São Carlos, São Paulo, Brazil). This novel approach to studying neurological disturbances has already been applied in hemodialysis patients. The technology is based on capturing small variations in the skull caused by ICP alterations through a voltage sensor in contact with the lateral region of the sagittal suture, providing real-time ICPw data. This ICPw is composed of three peaks (Figure 2a), and from the morphology of this wave, it is possible to infer intracranial compliance (Figures 2b – normal and 2c – altered).^6^

A first cross-sectional study using this technology in patients with ESKD undergoing hemodialysis indicated that these patients frequently experience alterations in intracranial compliance and that high-quality hemodialysis (according to Kt/V) might be effective in normalizing intracranial compliance.^7^ Kt/V is a formula used to measure dialysis adequacy, in which K is urea clearance by the dialyzer, t is the time of treatment, and V is the volume of urea distribution in the patient. The recommended Kt/V is maintained above 1.2 during hemodialysis.^8^ A subsequent follow-up study evaluated the intracranial compliance (by means of the ratio between the peaks P2/P1) pre-dialysis and post-dialysis, demonstrating that the P2/P1 ratio was less than that observed before the dialysis was done, reinforcing the previous finding that hemodialysis generates a positive effect on intracranial compliance.^9^

Based on these results, the need to investigate alterations in intracranial compliance in the initial stages of CKD as well as in those patients in stage 5 who do not undergo dialysis is required. Patients with CKD enter stage 5 when their GFR is < 15 mL/min/1.73 m^2^, but dialysis is usually indicated when the GFR is < 10 mL/min/1.73 m^2^.^10^

## OBJECTIVE

The aim of this study was to analyze intracranial compliance in different CKD stages (stages 1, 2, 3, 4, and 5 non-dialytic) compared with the results obtained in patients undergoing dialysis^9^ and a control group.

## METHODS

This study was conducted in two stages at a substitutive kidney therapy center (KTC) of a hospital in southern Brazil. Both protocols were approved by the Research Ethics Committee of Universidade Estadual de Ponta Grossa (protocols: 1.834.627, approved on 2016, Nov. 24; 4.039.453, approved on 2020, May 20) and the study followed the STROBE.^11^

### Participants

The participants were patients with chronic kidney disease from the southern region of the state of Paraná, most of whom have diabetes and high blood pressure and were being treated by the Unified Health System (in Portuguese, Sistema Único de Saúde – SUS). The first stage was an observational, longitudinal, prospective study of 42 patients with terminal kidney disease who underwent hemodialysis periodically, three times a week, for six months.^9^ The study started in January 2017 and ended in August 2018. The second stage was an observational, cross-sectional study that included 226 participants. Of those, 186 were patients with CKD in stages 1–5 but who still did not undergo any kind of substitutive kidney therapy, and 40 did not present with CKD and were classified as the control group. The choice of control group participants was randomized, including individuals of similar age groups as those in the CKD group. The second stage began in October 2019 and ended in October 2021. During both stages, the sampling was convenient because this was an unprecedented study according to the number of patients admitted at the KTC.

Every participant received information about the study and willingly participated after signing the two-part form of the Consent Term (Termo de Consentimento Livre e Esclarecido). As inclusion criteria, it was established that participants should be ≥ 18 years and considered legally capable. The clinical characteristics of patients were obtained using questionnaires and consultations with online medical records from the KTC. The study was conducted in accordance with the Code of Ethics of the World Medical Association (Declaration of Helsinki).

### Monitoring of intracranial compliance

Intracranial compliance was monitored using non-invasive equipment provided by the company Brain4care. Two examiners participated in the research, one for each stage, and they underwent previous training provided by the company. This method is innovative, validated,^12^ safe, and detects micrometric deformation of the cranial bones through a mechanical extensometer attached to a sensor. This sensor is fixed to a band and coupled to the lateral position of the patient’s head, approximately 2 cm above the ear. The detected deformations were transformed into electrical signals and displayed on a monitor. The amplification stage then occurs in which the equipment filters, amplifies, digitizes, and records the signals. The monitor displays are saved and sent to the Brain4care Analytics software, which provides us with a report of each display in which the wave morphology of the ICP and the amplitude of its peaks can be observed.^13^ The equipment used was properly calibrated by Brain4care.

At the moment of monitoring, the individuals stood still, sat down, and were monitored for approximately 3–5 min. The patients in the first stage, who underwent hemodialysis, were monitored pre- and post-dialysis in every session during the 6-month period in which they were assessed. Patients in the second stage underwent one monitoring session.

### Statistical analysis

Two comparison models were created for the P2/P1 ratio. The first included three groups: 1) individuals without CKD; 2) individuals with CKD who did not undergo substitutive treatment; and 3) individuals who underwent hemodialysis. The second model differs from the first because it includes three levels of CKD severity among patients with the disease who did not undergo hemodialysis. The levels of severity were: mild, which includes individuals at stages 1 and 2; moderate, which includes individuals at stages 3a and 3b; and severe, which includes individuals at stages 4 and 5. In this way, Model 2 included five groups: 1) individuals without CKD; 2) individuals with CKD in the mild form; 3) individuals with CKD the moderate form; 4) individuals with CKD in the severe form; and 5) individuals undergoing hemodialysis.

The average P2/P1 ratio of each patient undergoing hemodialysis was calculated pre- and post-dialysis monitoring. Pre-dialysis values were selected for a more reliable analysis, as post-dialysis values could be affected by all dialysis procedures.

Analysis of the quantitative value of the ICPw P2/P1 ratio in both models utilized an analysis of variance (ANOVA) with Tukey’s post-hoc test. We conducted the Shapiro-Wilk test to verify the normality of the data (P >0.05).

To assess the discriminative power of ICPw in CKD patients undergoing enhanced hemodialysis compared with those who still required it, we generated a receiver operating characteristic (ROC) curve using the P2/P1 ratio as a factor. The ROC curve plots the sensitivity (true positive rate) against 1-specificity (false positive rate) for different cut-off values of the P2/P1 ratio, displaying the relationship between sensitivity and specificity across a range of cut-off values. We calculated the area under the curve (AUC) to evaluate the overall diagnostic accuracy of non-invasive intracranial pressure measurements. The optimal cut-off value was determined by maximizing the Youden index, which combines sensitivity and specificity to measure the overall diagnostic accuracy. By comparing the ROC curves of patients with CKD not undergoing hemodialysis with those of patients with CKD undergoing hemodialysis, we assessed the discriminative power of non-invasive intracranial pressure measurements between the two groups. Statistical significance was set at a significance level of 5% (P < 0.05). All calculations were performed using GraphPad Prism version 9.00 Windows (GraphPad Software, La Jolla, California, USA).

## RESULTS

### Clinical parameters

The clinical parameters of the patients included in this study are shown in Table 1. The largest group we studied was patients with CKD undergoing hemodialysis (n = 42), and the smallest group was patients with stage 5 CKD not undergoing dialysis (n = 19). The age of the volunteers varied between 18 and 90 years.

**Table 1:** Mean age and percentage of individuals of each sex within each group (control group, stages 1–5 of chronic kidney disease who do not undergo hemodialysis, and stage 5 who undergo hemodialysis)

Clinical parameter	n	Age, in years, mean (range)	Sex, n (%)	
			Men	Women
Control group	40	45.0 (23–90)	45%	55%
CKD Stage 1	26	39.6(18–66)	35%	65%
CKD Stage 2	33	49.4 (26–73)	39%	61%
CKD Stage 3a	34	66.3 (28–78)	53%	47%
CKD Stage 3b	39	63.1 (36–81)	39%	61%
CKD Stage 4	35	64.54 (35–90)	43%	57%
CKD Stage 5 non-dialysis	19	60.6 (41–79)	47%	53%
CKD Stage 5 dialysis	42	55.8(21–87)	55%	45%

CKD = chronic kidney disease.

### Comparative analysis of the P2/P1 ratio results between the groups

**Figure 3:**
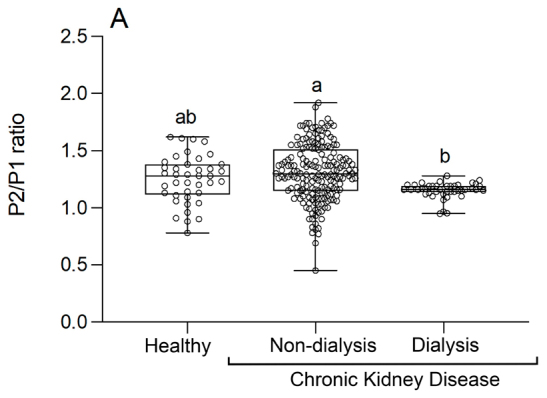
Comparison of the intracranial pressure P2/P1 ratio values of control participants, patients with chronic renal failure who do not undergo hemodialysis, and patients who undergo hemodialysis.

There was a significant difference between the analyzed groups (Figure 3); the patients undergoing dialysis mostly presented a P2/P1 ratio close to 1 (ratio P2/P1 ≤ 1 = normality). The group of patients not undergoing dialysis had the highest P2/P1 ratio. An alteration in intracranial compliance was also observed in the control group (healthy); the average P2/P1 ratio in these patients was > 1.

Box plot with the average and interquartile break of non-invasive intracranial pressure values obtained from healthy volunteers and patients with different stages of chronic kidney disease without and with hemodialysis. (A) ratio P2/P1, significant difference (P = 0.0002). Different letters represent significant differences (P < 0.05). The circles represent volunteers. Analysis of variance (ANOVA) with Tukey’s post hoc test

**Figure 4:**
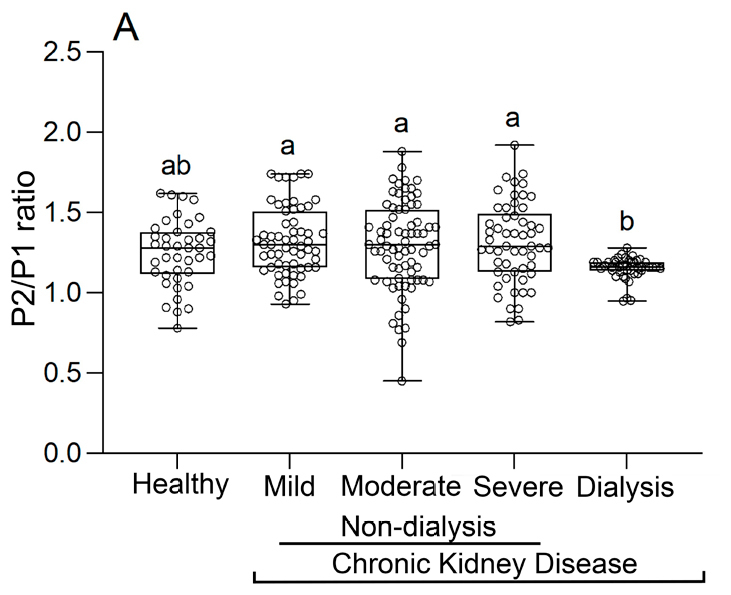
Comparison of the ICP P2/P1 ratio values of control participants, patients with chronic renal disease in mild, moderate, and severe stages who do not undergo hemodialysis, and patients who undergo hemodialysis.

Legend: Box plot showing the average and interquartile interval of non-invasive intracranial pressure values obtained from healthy volunteers and patients at different stages of chronic kidney disease. (A) ratio P2/P1, significant difference (P = 0.0018). Comparison between the groups: Different letters represent significant differences (P < 0.05). The circles represent volunteers. Analysis of variance (ANOVA) with Tukey’s post hoc test

The graph ahead (Figure 4) brings a wider analysis of the results presented in the previous graph (Figure 3). The patients in the group “without hemodialysis” were classified into three categories: mild CKD, moderate CKD, and severe CKD, for a thorough analysis of what happens during intracranial pressure in the different stages of kidney disease.

The mild, moderate, and severe CKD groups had similar P2/P1 ratios. However, there was a significant difference between the CKD groups without hemodialysis and patients undergoing hemodialysis. The healthy patients had similar results to the patients in the groups “mild,” “moderate,” and “severe,” undergoing hemodialysis.

**Figure 5:**
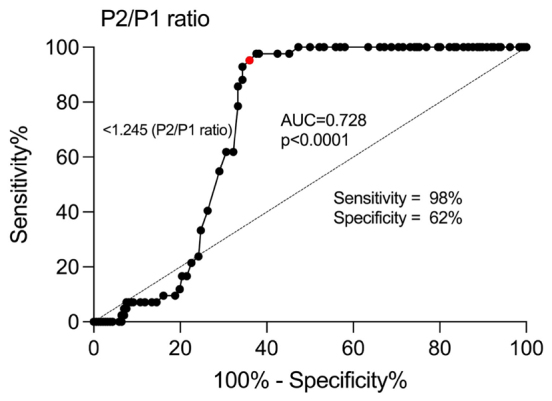
Receiver operating characteristic (ROC) curve of non-invasive intracranial pressure (P2/P1 ratio) in hemodialysis and non-hemodialysis patients with chronic kidney disease.

Legend: The ROC curve of the P2/P1 ratio was used to assess the distinguishing power between patients with CKD undergoing hemodialysis and not undergoing hemodialysis. The area under the curve (AUC): 0.728, sensitivity: 98%, specificity: 62%, cut-off: 1.245 (red dot), P < 0.0001.

An ROC curve was generated for the P2/P1 ratio to assess its diagnostic performance in patients with CKD in both the hemodialysis and non-hemodialysis groups. The P2/P1 ratio demonstrated discriminative power, with an AUC value of 0.728 (P < 0.0001). Hence, the P2/P1 ratio serves as a good marker for distinguishing between hemodialysis and non-hemodialysis patients with CKD (Figure 5).

## DISCUSSION

In this study, we demonstrated that the group of individuals undergoing hemodialysis presented better intracranial compliance than the group of individuals with CKD who did not undergo substitutive kidney therapy. One of the most affected organs by CKD is the brain, and varied neurological damages are commonly observed in kidney patients, such as cognitive deterioration, encephalopathy, convulsions, and strokes.^14^ One of the pathological mechanisms is brain edema, which can be due to the accumulation of uremic toxins^15^ and increase in extracellular fluid, progressing as the GFR decreases, causing ICP elevation.^16^ Patients who undergo hemodialysis are in the terminal stage of the disease and present a very low GFR. In addition, the ICP in these patients was better, probably because of the decrease in fluid volume due to hemodialysis. This aligns with our results of the ICPw comparison pre- and post-dialysis, indicating improvement after the procedure.^9^ Therefore, hemodialysis plays an important role in the improvement of ICPw. The mild, moderate, and severe CKD groups exhibited similar P2/P1 ratios. The hypothesis for this result is the administration of adequate treatment for these patients and the differences in the protocols for each stage. As CKD progresses, the disease management protocol becomes more complex and rigid, requiring better control of laboratory parameters and administration of medications.^10^

An additional interesting finding of this study was the distinction between the groups of patients with CKD who underwent hemodialysis and those who did not. Using the ROC curve, we determined a cut-off point of 1.245 for the P2/P1 parameter, with an AUC value of 0.728 and a sensitivity of 98%. AUC is a widely used performance measure for assessing the accuracy of a binary classification model. AUC values close to 1.0 indicate excellent performance in correctly classifying true categories; values between 0.7 and 0.9 are considered indicative of moderate to good performance; and AUC values between 0.5 and 0.7 are deemed poor to moderate performance.^17^ Hence, our findings suggest that the P2/P1 ratio serves as a reliable marker for distinguishing between the two groups.

Neurological manifestations may appear in any CKD stage, with higher chances in the late stages.^18^ In the early stages, the main symptoms reported are: difficulty focusing, lack of attention, emotional unbalance, depression, and recent memory impairment.^14^ In the more advanced stages, the accumulation of organic waste and toxins causes loss of consciousness, convulsions, and coma.^15^ Although the neurological symptoms become more evident in the terminal stage of the disease, their identification and adequate management in the early stages delay the progression and effects of these complications.^19^ Usually, initiating dialysis in patients with chronic kidney disease is determined based on the presentation of typical symptoms of kidney insufficiency, which often occur with a GFR 5–10 mL/min/1.73 m^2^, and include mental confusion and loss of consciousness.^20^ In this context, the monitoring of the ICPw might aid in investigating neurological disturbances at every stage of CKD. In summary, the ICPw follow-up in the last CKD stage might be one of the determining factors in initiating substitutive renal therapy. Hemodialysis has demonstrated efficient ICPw improvement in these patients, reducing the symptoms and improving the quality of life.

In the control group, a large number of individuals presented with alterations in ICPw. This may be attributed to the presence of diseases such as arterial hypertension, diabetes, and obesity observed in some of these patients, which may interfere with brain self-regulation. Brain self-regulation corresponds to the capacity of the brain to maintain adequate blood flow through variations in arterial pressure,^21^ and its limits are an average arterial pressure between 60 and 160 mmHg for healthy adults.^22^ Furthermore, thickening of the internal basal membrane of the brain microvasculature and an increase in the P2 peak in mice with diabetes was observed. The hypothesis is that the damage to the brain microvasculature in diabetes mellitus compromises intracranial compliance.^23^ In addition, diabetic ketoacidosis, a complication of diabetes, may lead to brain edema, which may cause intracranial hypertension.^24^ Moreover, many authors associate obesity with the development of idiopathic intracranial hypertension.^25,26^ Idiopathic intracranial hypertension is characterized by increased intracranial pressure of uncertain etiology, primarily affecting obese women of reproductive age.^27^ Although its pathophysiological mechanism is unknown, the risk of developing idiopathic intracranial hypertension intensifies according to the body mass index.^25,28^ Notwithstanding, overweight and obesity are factors that increase the probability of cerebrovascular diseases, which may lead to intracranial hypertension.^29^ Other possibilities for the ICPw alteration in patients of the control group could include undiagnosed medical conditions that may affect intracranial compliance and arterial hypertension, which may exceed the brain’s self-regulation limit. Although these data are noteworthy, they are not the focus of this study, and more studies are being conducted with the objective of clarifying the behavior of the ICPw and related variables.

This research has some limitations, such as convenience sampling of only one KTC, which may generate biased selection. Furthermore, in the second stage, only one monitoring of each patient was performed, providing accurate data without the possibility of following up. Additionally, both stages were not performed concurrently, with the possibility of protocol changes between the years of the study.

Despite its limitations, this study demonstrates the relevance of monitoring ICPw in patients with CKD from the early stages until the terminal stage. The incorporation of the non-invasive monitoring method of the ICPw in the follow-up of patients with chronic renal diseases aids in their treatment, resulting in an improved quality of life and preventing complications such as strokes, which are common in these individuals. In patients in the terminal stage, ICPw monitoring may be an auxiliary tool in the decision to initiate hemodialysis, as it influences ICPw improvement.

## CONCLUSION

In conclusion, hemodialysis influences ICPw control in patients with CKD at the terminal stage. In addition, we suggest non-invasive monitoring using ICPw as a new parameter in deciding the moment at which the patient with CKD must initiate substitutive kidney therapy, with the objective of minimizing the risks to the brain and improving the patient’s quality of life.

## References

[1] Kovesdy CP (2022). Epidemiology of chronic kidney disease: an update 2022. Kidney Int Suppl.

[2] Bello AK, Okpechi IG, Osman MA (2022). Epidemiology of hemodialysis outcomes. Nat Rev Nephrol.

[3] Levey AS, Eckardt KU, Tsucamoto Y (2005). Definition and classification of chronic kidney disease: A position statement from kidney disease: Improving Global Outcomes (KDIGO). Kidney Int.

[4] Zwang N, Nigwekar S, Steele D, Magee C, Tucker J, Singh A (2016). Core concepts in dialysis and continuous therapies.

[5] Rizzo MA, Frediani F, Granata A (2012). Neurological complications of hemodialysis: state of the art. J Nephrol.

[6] Moraes FM, Rocha E, Barros FCD (2022). Waveform morphology as a surrogate for ICP monitoring: a comparison between an invasive and a noninvasive method. Neurocrit Care.

[7] Rickli C, Kalva DC, Frigieri GH (2022). Relationship between dialysis quality and brain compliance in patients with end-stage renal disease (ESRD): a cross-sectional study. São Paulo Med J.

[8] Vanholder R, Glorieux G, Eloot S (2015). Once upon a time in dialysis: the last days of Kt/V?. Kidney Int..

[9] Rickli C, Cosmoski LD, Santos FA (2021). Use of non-invasive intracranial pressure pulse waveform to monitor patients with end-stage renal disease (ESRD). PLoS One.

[10] Brasil (2014). Diretrizes clínicas para o cuidado ao paciente com doença renal crônica – DRC no sistema único de saúde..

[11] STROBE Statement. Strengthening the reporting of observational studies in epidemiology.. What is strobe?.

[12] Cabella B, Vilela GHF, Mascarenhas S (2016). Validation of a new noninvasive intracranial pressure monitoring method by direct comparison with an invasive technique. Acta Neurochir Suppl.

[13] Ballestero MFM, Frigieri G, Cabella BCT, Oliveira SM, Oliveira RS (2017). Prediction of intracranial hypertension through noninvasive intracranial pressure waveform analysis in pediatric hydrocephalus. Childs Nerv Syst.

[14] Jabbari B, Vaziri ND (2018). The nature, consequences, and management of neurological disorders in chronic kidney disease. Hemodial Int.

[15] Hamed SA (2019). Neurologic conditions and disorders of uremic syndrome of chronic kidney disease: presentations, causes, and treatment strategies. Expert Rev Clin Pharmacol.

[16] Varela AM, Pecoits Filho RFS (2006). Interações entre a doença cardiovascular e a doença renal crônica. J Bras Nefrol.

[17] Carter JV, Pan J, Rai SN, Galandiuk S (2016). ROC-ing along: Evaluation and interpretation of receiver operating characteristic curves. Surgery.

[18] Lakshman SG, Ravikumar P, Kar G (2016). A comparative study of neurological complications in chronic kidney disease with special reference to its stages and haemodialysis status. J Clin Diagn Res.

[19] Arnold R, Issar T, Krishnan AV, Pussell BA (2016). Neurological complications in chronic kidney disease. JRSM Cardiovasc Dis.

[20] InformedHealth.org [Internet]. (2018). Chronic kidney disease: When is the best time to start dialysis?.

[21] Petersen LG, Ogoh S (2019). Gravity, intracranial pressure, and cerebral autoregulation. Physiol Rep.

[22] Armstead WM (2016). Cerebral blood flow autoregulation and dysautoregulation. Anesthesiol Clin.

[23] Onodera H, Oshio K, Uchida M, Tanaka Y, Hashimoto T (2012). Analysis of intracranial pressure pulse waveform and brain capillary morphology in type 2 diabetes mellitus rats. Brain Res.

[24] Szmygel Ł, Kosiak W, Zorena K, Myśliwiec M (2016). Optic nerve and cerebral edema in the course of diabetic ketoacidosis. Curr Neuropharmacol.

[25] Subramaniam S, Fletcher WA (2017). Obesity and weight loss in idiopathic intracranial hypertension: a narrative review. J Neuroophthalmol.

[26] Hannerz J, Ericson K (2009). The relationship between idiopathic intracranial hypertension and obesity. Headache.

[27] Thurtell MJ (2019). Idiopathic intracranial hypertension. Continuum.

[28] Mollan SP, Ali F, Hassan-Smith G (2016). Evolving evidence in adult idiopathic intracranial hypertension: pathophysiology and management. J Neurol Neurosurg Psychiatry.

[29] Letra L, Sena C (2017). Cerebrovascular disease: consequences of obesity-induced endothelial dysfunction. Adv Neurobiol.

